# Detection of Vaginal Metabolite Changes in Premature Rupture of Membrane Patients in Third Trimester Pregnancy: a Prospective Cohort Study

**DOI:** 10.1007/s43032-020-00338-9

**Published:** 2020-10-06

**Authors:** Lou Liu, Han-Jie Xu, Jia-Le Chen, Zhong Chen, Hui-Ying Zhan, De-Xiang Xu, Yu Chen, Zheng-Feng Xu, Dao-Zhen Chen

**Affiliations:** 1 The Affiliated Wuxi Maternity and Child Health Care Hospital of Nanjing Medical University, Wuxi, Jiangsu China; 2 Nanjing Medical University, Nanjing, China; 3 Anhui Medical University, Hefei, Anhui China; 4 Research Institute for Reproductive Health and Genetic Diseases, The Affiliated Wuxi Maternity and Child Health Care Hospital of Nanjing Medical University, Wuxi, Jiangsu China; 5 Department of Prenatal Diagnosis, Nanjing Maternity and Child Health care Hospital, Women’s Hospital of Nanjing Medical University , Nanjing, Jiangsu China

**Keywords:** Premature rupture of membranes, Metabolomics, Vaginal dysbiosis, Vaginal microbiome, Asymptomatic vaginitis

## Abstract

Premature rupture of membranes (PROM) is usually associated with pregnant and neonatal complications. Most of the PROM cases are caused by ascending asymptomatic genital infection. In China, PROM (15.3%) is more common than spontaneous preterm labor (7.3%) and leads to more adverse pregnancy outcomes. Here, we designed a prospective cohort study to measure the metabolomics changes in vaginal swab samples and explored their potential contribution to PROM. A total of 260 differentially expressed metabolites were identified and further analyzed. In the PROM group, N-acetyl-d-galactosamine and sucrose were downregulated (*P =* 0.0025, *P =* 0.0195, respectively), both of which are the upstream metabolites of the glycolysis pathway. Furthermore, estriol 3-sulfate 16-glucuronide (*P =* 0.0154) and 2-methoxy-17beta-estradiol 3-glucosiduronic acid (*P =* 0.004), two final metabolites in steroid hormone biosynthesis, were both downregulated in the PROM group. Finally, we found two catechin metabolites (epigallocatechin-7-glucuronide, *P =* 0.0009; 4′-methyl-epigallocatechin-7-glucuronide, *P =* 0.01) as well as DL-citrulline (*P =* 0.0393) were also significantly downregulated in the PROM group compared with the healthy control (HC) group, which are related to important antioxidant and anti-inflammatory activities in the human body. Altogether, metabolite changes in glycolysis, steroid hormone biosynthesis, and antioxidant/anti-inflammatory pathways may contribute to (or be a consequence of) vaginal dysbiosis and PROM. Metabolite pathway analysis is a new and promising approach to further investigate the mechanism of PROM and help prevent its unfavorable pregnant outcomes at a functional level. Trial registration number: ChiCTR2000034721

## Introduction

Premature rupture of membranes (PROM) is defined as spontaneous rupture of membrane occurring before the onset of labor, which is often accompanied by ascending genital infection [1], excessive antibiotic administration, fetal infection, hydramnios, microbial invasion to the amniotic cavity [2], and a higher rate of cesarean section [3, 4]. Preterm premature rupture of membrane (pPROM) is defined as PROM happening before 37 gestational weeks, which is associated with about 30% preterm labor [5]. Many studies have reported that vaginal microbiome dysbiosis is a major cause of pPROM [1, 6]. Serrano et al. [7] reported the rate of preterm birth (PTB) is about 10% in the USA, and the risk of PTB is higher in the African-American than that of European ancestry population [8]. According to GENOVESE’s study [9], premature rupture of membranes (PROM) affects 8–10% of all pregnancies. So the incidence of PTB is slightly higher than PROM; however, it is opposite in China which may reflect the population difference. Xia, H., et al. [10] reported that the incidence of PROM in China is 15.3% in recent years. In contrast, the weighted nationwide incidence of PTB was 7.3% in China from 2015 to 2016 [11], much lower than that of PROM in China in 2015. Thus, it is critical to further study the causes of PROM in the Chinese population and develop a new clinical examination methodology to prevent PROM from happening.

Based on previous studies, *Candida albicans*, *Trichomonas*, and *Gardnerella* are the common pathogens found in mixed vaginitis and patients are usually treated with fluconazole or metronidazole. Their symptoms may be relieved after the treatment; however, the vaginitis often recur during pregnancy, and sometimes even more than twice [12]. Furthermore, most of the Chinese PROM patients may have neither clinical symptom nor positive pathogen test results in vaginal discharge. In other words, an apparent feature of PROM in China is those patients have no clinical symptoms such as increased leucorrhea, vulva titillation, or positive pathogen test result so that they do not receive treatment timely before they encounter PROM. Supporting this, a previous study also showed that about 30–50% of vaginal infection did not have clinical complications [13]. The main cause of PROM is ascending genital infection, which results in weakening membrane tension and the rupture of membranes [14]. Besides, hormone change in pregnancy may also contribute to vaginitis and its recurring. Furthermore, it has drawn serious attention in recent years that certain types of mixed vaginal infection causing vaginal dysbiosis [15] cannot be diagnosed with culture approach so that the infection cannot be treated timely and effectively.

As one of the culture-independent techniques, the 16S metagenomics approach has been developed to evaluate vaginal microbiome composition in pregnant women and it can identify much more pathogen species in pregnant women than before. *Mobiluncus*, *Leptotrichia*, *Ureaplasma*, and *Mycoplasma* are most commonly detected in these pregnant women’s vaginal swabs [16]. The culture-independent technique is also very helpful in the diagnosis of aerobic vaginitis, especially in BV, which has a strong relation to PROM and PTB [17].

Vaginal metabolites are the final downstream products of all the biochemical processes and the products of interaction between the mucous epithelium of the vagina and pathogens, so it can more closely reflect the phenotype at a functional level than vaginal microbiome composition. Therefore, metabolomics is a promising technique to figure out early genital infection during pregnancy period so that targeted treatment can be carried out to address asymptomatic genital infection, protect a healthy vaginal microbiome composition, and thus prevent PROM at from the very beginning. Vaginal metabolites testing has drawn more and more attention recently [18]. Our current study focused on the vaginal metabolite change of PROM women in the Chinese population and explored the relationship between PROM and vaginal metabolites in pregnant women of 31–36 gestational weeks. Our study has identified some small molecular metabolite and metabolic pathway changes that are related to PROM and these markers can be applied as an indication of vaginal microbiome dysbiosis in the early stage. This test can be easily performed to support accurate diagnosis of vaginal dysbiosis and improve PROM pregnancy outcomes.

## Materials and Methods

### Study Design and Subject Recruitment

This study was designed as a clinical cohort study. The recruitment criteria included pregnant women (singleton, head presentation) who received regular prenatal examination, and then experienced vaginal labor in the obstetric department of The Affiliated Wuxi Maternity and Child Health Care Hospital of Nanjing Medical University from 2019 to 2020. Exclusion criteria were pregnant complications which may disturb the study including preeclampsia, gestational diabetes, malnutrition, multiple gestation, breech presentation, preterm labor experience, short cervical length, preterm labor history, oral or vaginal medicine administration within 48 h before admission, sexual activity within 48 h before admission, and vaginal douching within 48 h before getting swab.

### Ethical Approval

Recruitment and sampling procedures were performed under the Declaration of China and applicable local regulatory requirements after approval from the Ethics Committee of The Affiliated Wuxi Maternity and Child Health Care Hospital of Nanjing Medical University (2019-02-0402-01). Written informed consent was obtained from all the subjects included in this study. The privacy rights of all these subjects were strictly observed and protected.

### Sample Collection and Process

Totally, 163 women were recruited in this study. Vaginal swabs were collected at the posterior fornix between 31 and 36 gestational weeks, and sampling swabs were stored at − 80 °C within 5 min. Finally, 36 women among the study cohort developed PROM. A group of 10 PROM women was selected randomly and a group of 10 women without PROM (healthy control, HC) was selected randomly. Their swab samples were subjected to untargeted liquid chromatography (LC) coupled with tandem mass spectrometry (LC-MS/MS) based metabolomics analysis by BGI Genomics, China.

In brief, vaginal swabs were weighed and eluted in methanol-water (1:1) to a final concentration of 50 mg vaginal fluid/ml. Samples were then microfiltered and passed through a Agilent 1290 Infinity HPLC coupled to Q-Exactive Orbitrap mass spectrometer (Thermo Fisher Scientific). All samples were analyzed by full MS scanning between the ranges of 50–750 m/z in both positive ion and negative ion mode at 140000 sharpness of separations. A separate LC-MS method was performed for the relative quantification with increased sensitivity.

### Data Processing, Metabolomics, and Clinical Data Analysis

The raw MS data was exported to and further analyzed with metaX. Metabolites were identified and annotated with Compound Discoverer 3.0 (Thermo Fisher Scientific, USA) through BGI, mzCloud, and Chemspider libraries. Differentially expressed metabolites were filtered with PLS-DA model (VIP ≥ 1) and those with fold change ≥ 1.2 (or ≤ 0.83) and *P* value < 0.05 (Student’s *t* test) were considered a significant difference between PROM and HC groups. Differentially expressed metabolite heatmap and metabolic pathway enrichment bubble chart (against KEGG database) were plotted with MetaboAnalyst web tools.

Clinical characteristic data was analyzed with the unpaired non-parametric Wilcoxon-Mann-Whitney *U* test and one-way variance (ANOVA) in SPSS 20.0 to check if there is a statistical significant difference between PROM and HC groups.

## Results

### Demographical and Clinical Data

A total of 163 women were initially recruited in the study cohort; however, we failed to follow up 40 subjects due to the outbreak of COVID-19. Among the 123 subjects left in the study, 36 women encountered PROM. The incidence of PROM in this study was 29.3%, and significantly higher than the previous report [10], which was probably due to the different diagnosis standards we had applied. We typically comply with the guidelines from “ACOG Practice Bulletin No. 188: Prelabor Rupture of Membranes” for PROM diagnosis, under which women who only have limited amniotic fluid flowing out of the vagina (with fetal fibronectin test positive) are all diagnosed as PROM [19]. The 20 study subjects, including 10 PROM and 10 HC subjects, were all of Han ethnicity in the Chinese population. Their clinical characteristics were collected and analyzed. There were no significant differences found between the two groups in maternal age (*P =* 0.23), nulliparity(*P =* 0.50), GA at sampling(weeks, *P =* 0.73), tocolysis treatment, GA at delivery (weeks, *P =* 0.33), latency from sampling to delivery (weeks, *P =* 0.17), birth weight (*P =* 0.83), and neonatal gender (*P =* 1.00). None of the study subjects received steroid admission or antibiotic treatment before vaginal sampling. Interestingly, the mean white blood cell (WBC) counts of the PROM group was slightly higher than that of the HC group, but the number difference did not reach a statistical significance between the two groups(*P =* 0.08). None of the subjects was found positive in genital pathogen culture at admission, although half of them (10 subjects) had PROM in the end. Only one subject in the PROM group received tocolysis treatment among the total 20 subjects (Table 1). Altogether, the clinical characteristics of the study subjects from two groups matched well.

**Table 1:** Clinical characteristics of the subjects

Characteristics	Pregnant women cohort (*n* = 163)			Metabolomics detection samples		
	PROM cases (*n* = 36)	Controls (*n* = 87)	Lost to follow-up (*n* = 40)	PROM cases (*n* = 10)	Controls (*n* = 10)	*P* value
Maternal age^a^	29.1 (22–39)	29.5 (19–40)	29.3 (21–41)	27.4 (22–32)	29.5 (22–36)	0.23^†^
Nulliparity	32	78	34	7	6	0.64^‡^
White blood cell at admission (× 10^9^/L)	8.6 (6.1–13.1)	8.1 (5.1–10.2)	–	9.0 (6.2–12.7)	8.0 (5.2–9.6)	0.08^†^
Positive genital cultures at admission	1	4	0	0	0	–
Gestational age at sampling (weeks) ^a^	33.3 (31.0–36.6)	33.5 (31.5–36.5)	33.3 (31.4–36.0)	33.9 (32.0–35.1)	34.2 (32.8–36.0)	0.73^†^
Steroid administration	0	0	0	0	0	–
Tocolysis treatment	1	1	–	1	0	0.30^‡^
Gestational age at delivery (weeks)^a^	38.4 (34.7–40.1)	39.4 (34.8–40.8)	–	38.9 (37.9–39.4)	38.7 (37.4–39.9)	0.33^†^
Latency from sampling to delivery (weeks)^a^	5.3 (0.5–7.8)	5.8 (2.3–8.3)	–	5.3 (3.2–7.1)	4.5 (3.1–7.6)	0.17^†^
Baby weight at birth (g)^a^	3375 (2640–4200)	3369 (2500–4500)	–	3210 (2960–3700)	3180 (2760–3780)	0.83^†^
Baby gender (male/female)	16/20	35/52	–	4/6	5/5	0.65^‡^

*PROM*, premature rupture of membranes

^a^Values are presented as median (mix-max)

^†^Independent-sample *T* test

^‡^Chi-square test

### Vaginal Metabolite Diversity

**Fig. 1:**
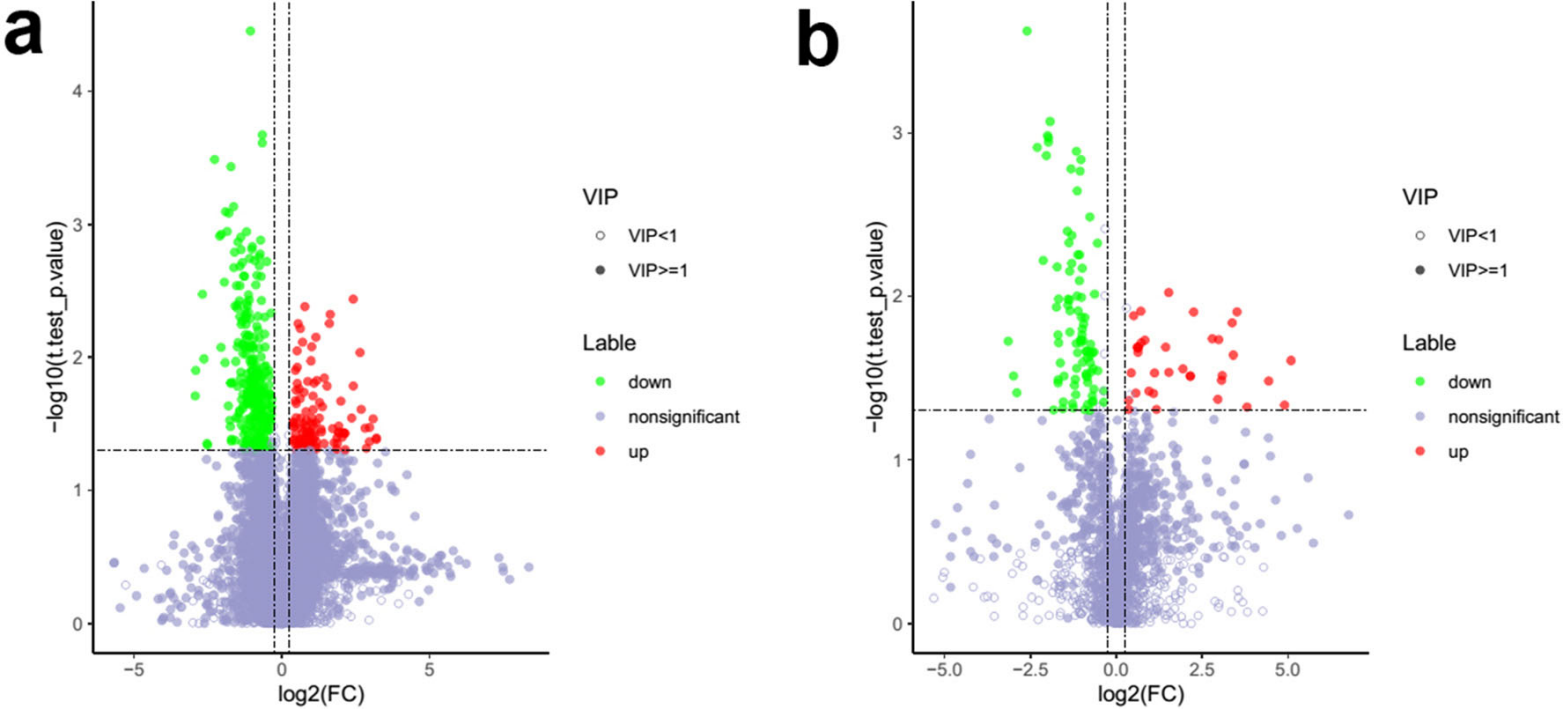
Volcano map of vaginal metabolites detected in the swap samples. A total 4718 vaginal metabolites were detected in positive mode (**a**) and 1339 vaginal metabolites were detected in negative mode (**b**). Metabolites with fold change ≤ 0.8333 and *P* value < 0.05 were considered downregulated metabolites and plotted in green dots. Metabolites with fold change ≥ 1.2 and *P* value < 0.05 were considered upregulated metabolites and plotted in red dots. VIP, variable important for the projection. Metabolites with VIP > 1 were considered having a significant contribution

All the mass spectrometry data were analyzed in metaX to determine metabolites’ molecular weight and abundance differences with Compound Discoverer 3.0 (Thermo Fisher Scientific, USA) in BGI Library, MzCloud, and Chemspider. PLS-DA (partial least-squares method-discriminant analysis), together with fold change, and Student’s *t* test was used to screen differentially expressed metabolites. *T* test was used to compare the metabolite expression level between the two groups (*P <* 0.05 was considered an indication of the significant difference). In the positive ion mode, we found 112 metabolites were upregulated and 286 metabolites were downregulated in the PROM group compared with the HC group. In the negative ion mode, we found 35 metabolites were upregulated and 91 metabolites were downregulated in the PROM group compared with the HC group (Fig. 1).

**Fig. 2:**
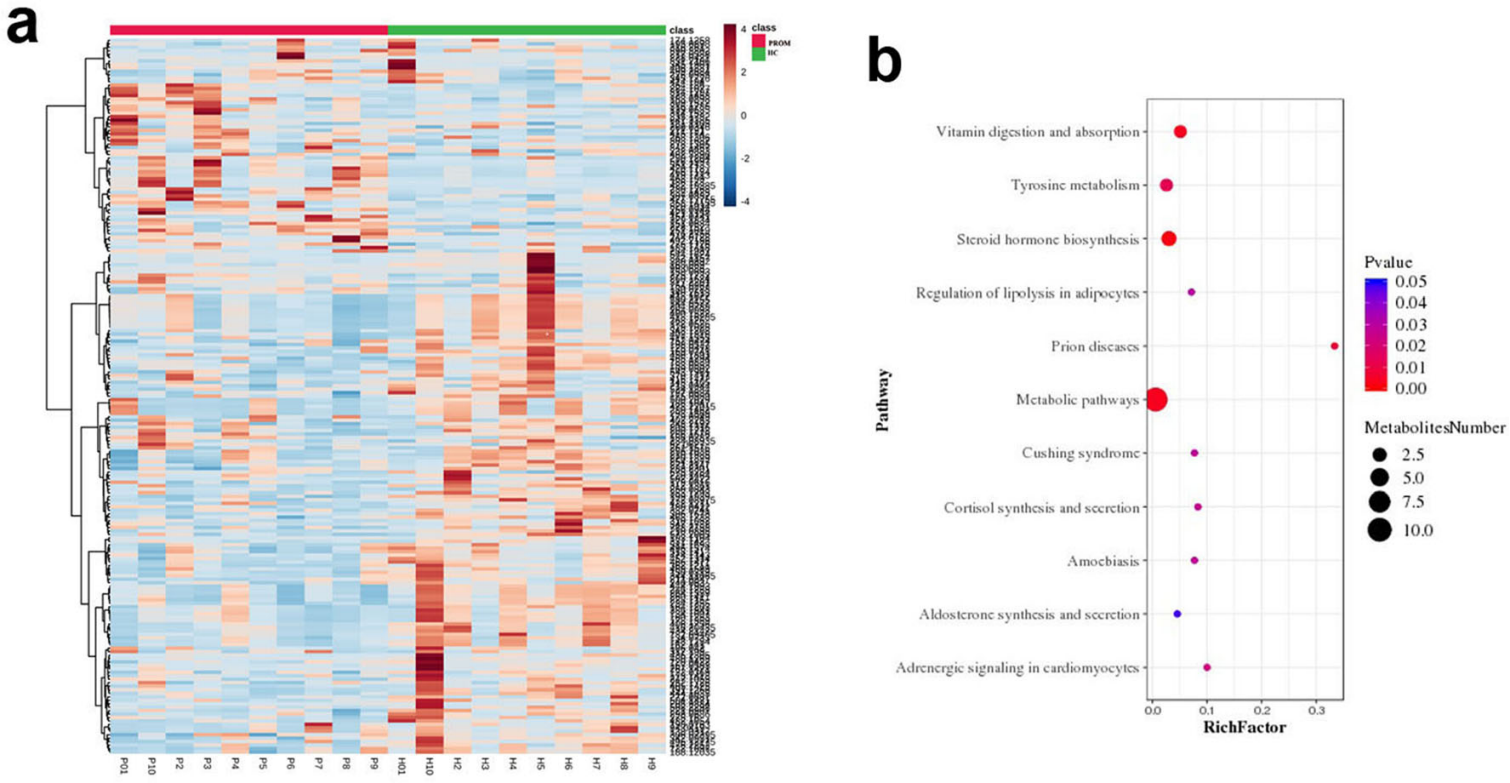
Differentially expressed metabolites heatmap and pathway enrichment. **a** Heatmap of all 260 annotated differentially expressed metabolites based on Compound Discoverer 3.0 in BGI, MzCloud, and ChemSpider libraries, including acid, lipid, glucose, and other metabolites. **b** These 260 metabolites enriched in multiple KEGG pathways with “metabolic pathways” and “steroid hormone biosynthesis” on the top

Because some metabolites could not be located in BGI Library or correctly annotated, they were filtering out during data processing. After this refining process, a total of 260 vaginal metabolites were identified in the PROM group and the HC group, including those that showed either significantly upregulated or significantly downregulated (Fig. 2a). We further applied Kyoto Encyclopedia of Genes and Genomes (KEGG) pathway enrichment analysis on these 260 metabolites. Interestingly, two pathways—metabolic pathway and steroid hormone biosynthesis—showed up with the most significant scores (Fig 2b). In the next parts, we focused on 4 metabolites which were related to the two metabolism pathways for further analysis.

### Downregulation of Glycolysis Pathway in the PROM Group

**Fig. 3:**
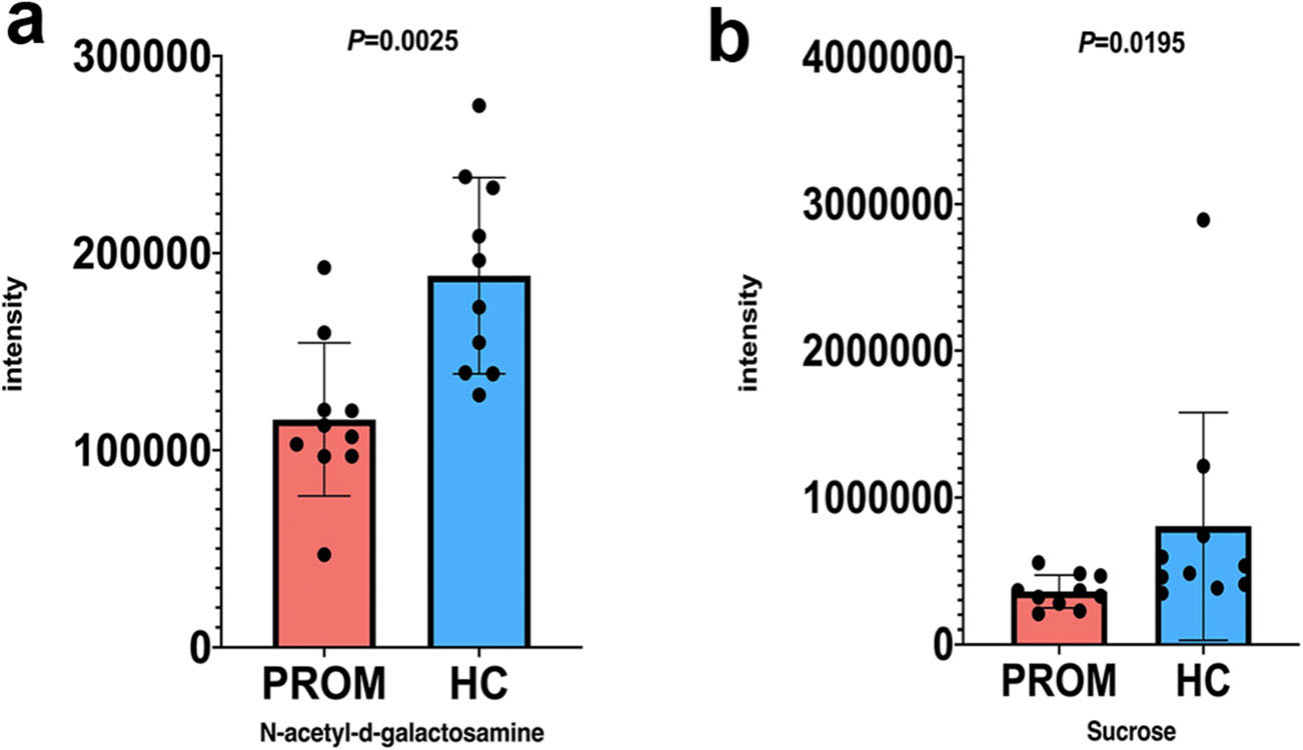
GalNAc and sucrose associated with the glycolysis/gluconeogenesis pathway were downregulated. **a** GalNAc was downregulated in PROM subjects (115,596) vs. HC subjects (188,508), *P =* 0.0025. **b** Sucrose was downregulated in PROM subjects (358,919) vs. HC subjects (805,223), *P* = 0.0195

To figure out how these differentially expressed metabolites were related and contributed to vaginal dysbiosis, we clustered metabolites based on species including acid metabolites, lipid metabolites, carbohydrate metabolites, respectively, and searched them against the KEGG database. Interestingly, N-acetyl-d-galactosamine (GalNAc) and sucrose are the upstream (or source) metabolites of glycolysis pathway and both of them were downregulated in the PROM group significantly in our study (*P =* 0.0025 and *P* = 0.0195, respectively). GalNAc and sucrose downregulation in PROM group probably meant that the glycolysis pathway was not fully fueled and those *Lactobacillus* microbes normally existing in a healthy vagina might not be able to produce sufficient lactic acid to maintain correct vaginal PH environment (Fig. 3, and further discussed below). Unfortunately, we were unable to detect different levels of lactic acid expression between PROM and HC groups in this metabolomics study (data not shown), probably due to the limited sample size. On the other hand, our swap sampling time might be at the early onset of vaginal dysbiosis so that the vaginal lactic acid level had not reached a significant difference.

### Downregulation of the Steroid Hormone Biosynthesis Pathway in the PROM Group

**Fig. 4:**
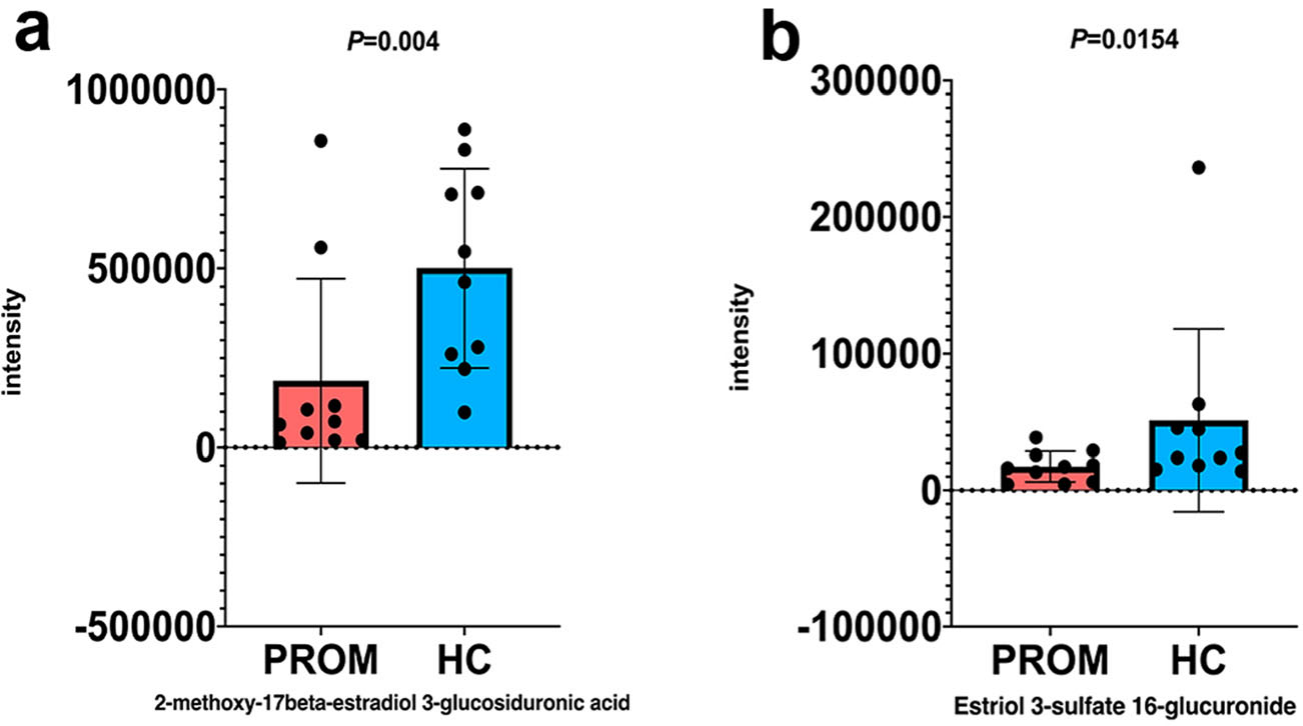
Two metabolites in the steroid hormone biosynthesis pathway were downregulated. **a** 2-methoxy-17beta-estradiol 3-glucosiduronic acid was downregulated in PROM subjects (186,898) vs. HC subjects (500,938), *P* = 0.0004. **b** Estriol 3-sulfate 16-glucuronide was downregulated in PROM subjects (17,378) vs. HC subjects (51,176), *P* = 0.0154

Besides these two glycolysis metabolites, we also found 2-methoxy-17beta-estradiol3-glucosiduronic acid (*P* = 0.004) and estriol3-sulfate16-glucuronide (*P* = 0.0154) were also downregulated in the PROM group. These two are both the final metabolites in the steroid hormone biosynthesis pathway (Fig. 4), and their low expression might lead to insufficient steroid biosynthesis in vaginal cells of PROM patients. Steroid hormones, such as progesterone and estrogen, play essential roles in maintaining a functional female reproductive system and successful pregnancy. Lack of sufficient steroid biosynthesis will inevitably result in loss of healthy vaginal microecology (discussed below).

### Downregulation of Antioxidant/Anti-inflammatory Activities in PROM

**Fig. 5:**
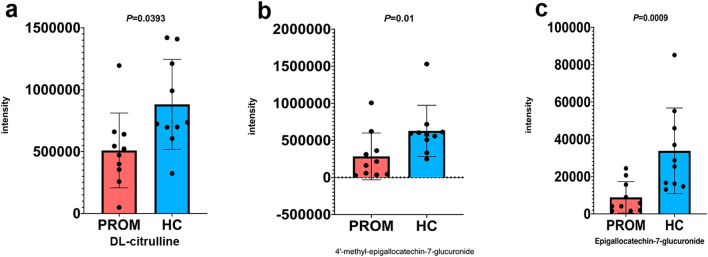
Three antioxidant metabolites were downregulated. **a** DL-citrulline was downregulated in PROM subjects (mean = 509,354) vs. HC subjects (mean = 881,660), *P* = 0.0393. **b** 4′-methyl-epigallocatechin-7-glucuronide was downregulated in PROM subjects (28,4251) vs. HC subjects (62,8426), *P* = 0.01. **c** Epigallocatechin-7-glucuronide was downregulated in PROM subjects (8878) vs. HC subjects (33,805), *P* = 0.0009

Lastly, we found three more metabolites that may be involved in antioxidant and anti-inflammatory activities in the human body including DL-citrulline (*P =* 0.0393), epigallocatechin-7-glucuronide (*P =* 0.0009), and 4′-methyl-epigallocatechin-7-glucuronide (*P =* 0.01) were significantly downregulated in the PROM group compared with the HC group (Fig. 5). Citrulline is a key metabolite in the arginine biosynthesis pathway and at the same time the precursor of nitric oxide (NO), an important antioxidant. On the other hand, catechin derivatives are also a class of important antioxidants and anti-inflammatory agents. Therefore, low expression of citrulline and catechin derivatives in the PROM group may be linked to impaired antioxidant and anti-inflammatory activities in the vaginal microenvironment and persistent infection (discussed below).

## Discussion

One of the key risk factors of PROM is the presence of bacterial, fungal, and mixed vaginal infections in the third trimester of pregnancy [20–22]. In general, vaginitis can be diagnosed by the Nugent score especially in BV [23] and pathogens such as group B streptococci are readily detected by culture approach [24] or microscope such as vaginal candidiasis [25]. However, the Nugent score usually cannot diagnose early-stage bacteria vaginitis. Also, the culture method may not be able to cover all species of pathogens [26]. Therefore, a large proportion of pregnant women who have been infected with anaerobic bacteria, mycoplasma, ureaplasma, etc. may not be successfully diagnosed by the culture method [27] and carry asymptomatic vaginitis at the early infection stage. Higher prevalence of asymptomatic vaginitis and abundance of bacterial vaginosis (BV)–related bacteria in these pregnant women [28] could bear increased risk of PROM.

In recent years, culture-independent approaches have been applied in analyzing and clustering pathogens [29]. Typically five *Lactobacillus* species dominate in the vagina to establish a healthy vaginal microorganism composition and maintain pH < 4.5 [30]. Studies have shown that if the healthy vaginal microecology is disrupted due to the change of vaginal microorganism composition, the morbidity of preterm birth and premature rupture of membranes increases [1, 9, 31]. When the severity of vaginal dysbiosis increases, it leads to the development of vaginitis and PROM as a result of the complicated interaction between pathogens and vaginal microecology [32]. We designed this study to focus on the differentially expressed metabolites between the PROM group and the HC group and tried to find out the impact of vaginal metabolites change on the development of PROM.

The asymptomatic vaginitis during pregnancy is caused by several factors, such as hormone change [33] and immunological tolerance against fetal antigens [34]. The clinical characteristics of the two groups in our study matched well, therefore, other factors that may disturb the cohort study outcomes, such as age, GA, and medical treatment received, were largely excluded. Interestingly, latency from sampling to delivery did not show a significant difference between the two groups, which suggested that most of the vaginal dysbiosis exists in the patients at a mild but persistent state, so that it did not develop quickly into acute genital infection and result in PROM. Supporting this, we noticed that most PROM pregnant women had neither clinical chief complaints related to bacteria or other pathogen infections nor pathogens identified during pregnancy physical check by culture method before PROM. In contrast, some pregnant women who were diagnosed with vaginitis such as BV and *Candida* vaginitis did not encounter PROM at all. Based on these facts, we deduced that vaginal dysbiosis is a result of the complicated interaction between pathogens and the vaginal epithelial immune system, and thus, there is no clear necessity between vaginal infection and PROM [31].

In our study, we found about 260 metabolites differentially expressed between PROM and Healthy Control (HC) groups with statistical significance, and most of them are acid metabolites. The heatmap did not show a centralized clustering feature and variations between individual samples were quite big. This feature suggested a mixed vaginal infection without any dominated species in our study subjects. This finding is consistent with a previous report showing that there is a 32.4% mixed infection in vaginal disease pregnant women at 28 gestational weeks [9]. To explore the underlying mechanism that results in these metabolites, we further applied KEGG pathway analysis in these differentially expressed metabolites (https://www.genome.jp/kegg/pathway.html), to figure out the relationship among them. We found two significant downregulated metabolites, including GalNAc (*P* = 0.0025) and sucrose (*P* = 0.0195), both of which were incorporated into the upstream of the glycolysis pathway.

Glycolysis was the first major metabolic pathway fully illustrated [35]. Bacteria can incorporate several sugars in the cytoplasm and utilize them for ATP production through glycolysis [36]. There are many types of sugars, including monosaccharides (glucose, fructose, mannose), disaccharides (sucrose, lactose, maltose), and amino sugars (glucosamine, N-acetylglucosamine), in the environment or host which can be used by bacteria. Different bacterial species use different types of sugars [37]. The pathway has many steps and regulation factors, for example, environment PH, the biofilm formation of bacteria, and enzyme activity. The glycolysis pathway ultimately leads to the catabolism of glucose and other hexoses into pyruvate [38, 39]. In a vaginal environment where oxygen supply is low, pyruvate is reduced to lactic acid [40], and this is a major source of lactic acid in the vagina to maintain PH < 4.5, favoring to maintain a balanced microbiome composition in the vagina to inhibit pathogenic bacteria proliferation. GalNAc and sucrose, two important upstream metabolites and a fuel source of the glycolysis pathway, were both detected low in the PROM group in our study. This observation may well reflect the scenario that proliferating pathogenic bacteria consume a large amount of sugar source in the vaginal dysbiosis microenvironment and leads to a low supply of GalNAc and sucrose to healthy *Lactobacillus* species. It further results in impaired lactic acid production and vaginal PH maintenance. The less acidic environment in turn favors aerobic bacteria to consume more sugars and proliferate more rapidly to develop into severe vaginitis.

In our study, we also found two steroid hormone biosynthesis pathway metabolites were downregulated in the PROM group. Cholesterol is the precursor of progesterone [41], which is the key steroid for the maintenance of pregnancy. Furthermore, progesterone modulates the maternal immune system favoring the tolerance toward fetal allograft [42]. Besides, it has been shown that progesterone increases the anti-inflammatory cytokine production in maternal T lymphocytes [43]. Furthermore, progesterone favors nitric oxide synthase activity and stimulates endothelial nitric oxide (NO) synthesis [44]. Progesterone also inhibits the activity of matrix-specific metalloproteases [45], which play an important role in the protection of fetal membranes. Estradiol (the predominant estrogen) is the downstream metabolite of progesterone in the steroid biosynthesis pathway [46]. The lack of estrogens leads to reduced vaginal epithelial cell glycogen secretion, which also results in the lack of lactic acid. Estrogens seem to affect both the infectivity of *Candida albicans* and the recruitment of host defenses [47]. Estradiol suppresses LPS-mediated apoptosis and promotes the anti-inflammatory TH2 responses in decidual stromal cells, thus contributing to a successful pregnancy as well as progesterone [30]. Altogether, disrupted steroid hormone biosynthesis pathway can facilitate pathogenic bacteria proliferation and weaken vaginal microecology’s immune system ability to clear pathogenic bacteria.

It is known that during the crosstalk between bacterial pathogens and host, the host cells can produce reactive oxygen species (ROS) and reactive nitrogen intermediates to fight against pathogens [48]. Vaginal epithelial cells also produce xanthine oxidase and superoxide dismutase, which can generate reactive oxygen intermediates, including H_2_O_2_ [49]. DL-citrulline is the metabolite of the arginine biosynthesis pathway and the precursor for nitric oxide (NO), an important antioxidant in the human body. On the other hand, catechin derivatives are flavons with high antioxidant potency and play an important role in response to oxygen stress [50]. In the microenvironment of asymptomatic mixed vaginitis patients, a low level of citrulline and catechin derivatives may impair the body’s antioxidant and anti-inflammatory response and lead to persistent infection and eventually the onset of PROM.

All of the metabolite changes discussed above may work together to impair the healthy vaginal microecology and lead to the development of vaginal dysbiosis and asymptomatic vaginitis. Interestingly, the ascending vaginitis can destroy the cervical fetal membrane through upregulating activation of matrix metalloproteinase-9 (MMP9), an enzyme involved in the remodeling of the fetal membranes and the cervix before the onset of labor, result in a weaken fetal membranes [51] and thus increase the risk of PROM during pregnancy.

It is worth mentioning that two research works using urinary metabolomics to study PROM and other perinatal conditions as well as asymptomatic infection have been reported recently [52, 53]. Interestingly, in one of the studies [52], the researchers found that lactic acid, erythritol, and ethanolamine levels were significantly higher in the pPROM group than in the PROM group, which indicated the existence of bacterial infection. We did not observe a statistically significant lactic acid change in our current study, which was likely due to the difference in sampling route, time, and disease stage. We will definitely consider using their sampling schedule in our follow-up study to further validate these metabolomics changes to assist in a more accurate diagnosis.

## Conclusions

Metabolomics is a powerful tool to profoundly understand the vaginal dysbiosis and study the mechanism of PROM caused by ascending asymptomatic vaginitis. Through metabolomics analysis, we have identified impaired glycolysis and lactic acid production pathway, steroid hormone biosynthesis pathway, as well as the antioxidant pathway in the PROM group subjects. These findings have partially explained the subtle changes in the vaginal microenvironment that may contribute to the development of vaginal dysbiosis, or otherwise be a consequence of the disease and serve as a biomarker for the diagnosis. As the next step, we will recruit more study subjects to further confirm our findings in a larger population. On the other hand, we have initiated 16S metagenomics analysis on these vaginal swaps to figure out the detailed changes of microbial composition in the PROM group, and will try to match the result with current metabolomics analysis data. Hopefully, with all these efforts, we will be able to use the new-generation biotech tools to monitor the onset of vaginal dysbiosis, guide the treatment of asymptomatic vaginitis, recover a healthy vaginal microecology, and thus prevent PROM from the very beginning.
